# Biotization of Endophytes *Trichoderma asperellum* and *Bacillus subtilis* in *Mentha spicata* Microplants to Promote Growth, Pathogen Tolerance and Specialized Plant Metabolites

**DOI:** 10.3390/plants11111474

**Published:** 2022-05-31

**Authors:** Dagoberto Castro-Restrepo, Maria Isabel Dominguez, Bertha Gaviria-Gutiérrez, Edison Osorio, Karina Sierra

**Affiliations:** 1 Unidad de Biotecnología Vegetal, Facultad de Ciencias Agropecuarias, Universidad Católica de Oriente, Cr46-40 B-50, Rionegro 054040, Colombia; mdominguez@uco.edu.co; 2 Grupo de Investigación Sanidad Vegetal, Facultad de Ciencias Agropecuarias, Universidad Católica de Oriente, Rionegro 054040, Colombia; sanidadveg.inv2@uco.edu.co; 3 Grupo de Investigación en Sustancias Bioactivas GISB, Facultad de Ciencias Farmacéuticas y Alimentarias, Universidad de Antioquia, Calle 70 No. 52-21, Medellín 050010, Colombia; edison.osorio@udea.edu.co (E.O.); karina.sierra@udea.edu.co (K.S.)

**Keywords:** spearmint, carvone, rosmarinic acid, total polyphenols, *Rhizoctonia* sp.

## Abstract

In the present study, the effect of biotization of *Mentha spicata* microplants with *Trichoderma asperellum* and *Bacillus subtilis* on growth, *Rhizoctonia* sp., incidence, and specialized metabolites content was evaluated. Analyses of root tissues of the microplants showed 100% endophytism with both microorganisms. During the acclimatization phase, plants with the endophytes *T. asperellum* and *B. subtilis* had a survival rate of 95% and 93%, respectively, compared to 75% for control plants. Then, under greenhouse conditions, a trial was carried out with biotized plants with or without *Rhizoctonia* sp. inoculation, plants inoculated with *Rhizoctonia* sp., and endophyte- and pathogen-free control. Biotized plants with the endophytes showed higher dry biomass and the incidence of *Rhizoctonia* was lower (8% for *T. asperellum* and 10% for *B. subtilis*) compared to plants inoculated with the pathogen (82%). In addition, plants with *T. asperellum* had the highest contents of total polyphenols (280 GAE/100 mg sample) and rosmarinic acid (28 mg RA/100 g sample). Thus, this study shows the potential of the technique of using the endophytes *T. asperellum* and *B. subtilis* on *M. spicata* microplants to improve plant survival and growth, decrease the incidence of *Rhizoctonia* sp., and improve the contents of specialized metabolites, which can contribute to the sustainable management of this crop.

## 1. Introduction

*Mentha spicata* L., known as spearmint, is used as an aromatic and spice plant [1]. Due to its phenolic compounds and terpenes such as rosmarinic acid and carvone, respectively, they are considered natural therapeutic alternatives in medical applications and functional foods [2,3,4]. Commercially, *M. spicata* is propagated vegetatively by stolons and cuttings; however, successive use of the same type of material has the risk of spreading diseases such as fungi, bacteria, and viruses [5]. One of the limiting diseases is caused by *Rhizoctonia solani*, which is a soil pathogen that causes various plant symptoms such as damping-off, stem and rhizome rot, among others [6]. In this sense, in vitro propagation techniques offer the alternative of producing quality materials free of pathogens [7].

Conventionally, micropropagation techniques are carried out under completely aseptic conditions. However, due to the current knowledge on the role of endophytic microorganisms as growth promoters and inducers of pest tolerance, importance is being given to their use in some of the stages of micropropagation such as proliferation, rooting and acclimatization, which is known as biotization [8].

Micropropagation processes take place in a highly protected environment without the possibility of interaction with microorganisms normally found in nature [9]. Therefore, the plants obtained are susceptible to biotic and abiotic stress problems during the acclimatization and development stage. Recent studies have attempted to re-establish the link between beneficial microorganisms and the in vitro multiplication process. These microorganisms can have a positive impact on the survival and growth of plantlets in the greenhouse and then in field conditions [10].

The most studied endophytic microorganisms are fungi and bacteria that complete all or part of their life cycle in living plant tissues and have great potential as sources of new natural products for exploitation in agriculture, medicine, and industry [11]. These organisms can have effects on plant survival such as protection against environmental stress and microbial competition [12]. Among them are *Trichoderma* spp. and *Bacillus* spp. which have been widely used as biocontrol agents and biostimulants to inhibit pathogen infection, induce systemic or localized resistance, enhance tolerance to biotic stresses, and improve plant vigor and growth [13,14]. Biotization of microplants is a promising biotechnological practice to improve seedling survival and quality during the acclimatization phase and, under field conditions, increase tolerance to biotic and abiotic stresses; therefore, it can contribute to the reduction of chemical inputs in plant production [15,16,17].

The aim of this study was the biotization of *M. spicata* microplants with *Trichoderma asperellum* and *Bacillus subtilis* to determine their level of endophytism and their effect on plant development, tolerance to *Rhizoctonia* sp., and the contents of total phenols, rosmarinic acid, and carvone under greenhouse conditions.

## 2. Results

### 2.1. Antagonism of T. asperellum and B. subtilis against Rhizoctonia sp.

*Rhizoctonia* sp. on mint crops is a pathogen with a necrotrophic life cycle that causes rotting and death of rhizomes, stems, and roots. From an isolate of the fungus, in vitro antagonism tests were carried out against the endophytes *T. asperellum* and *B. subtilis*.

**Figure 1:**
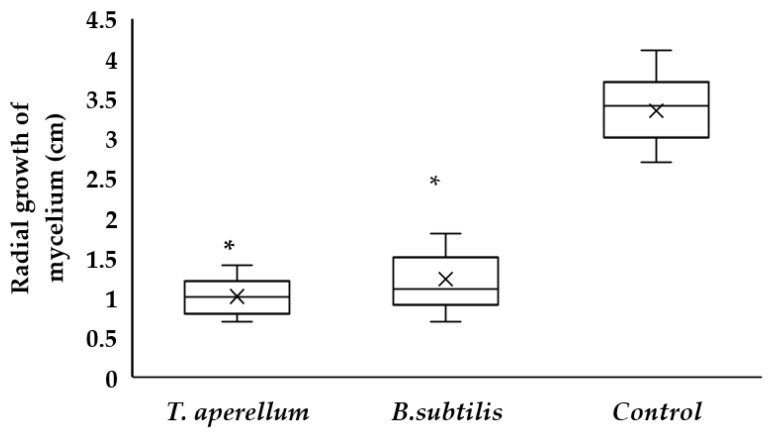
Radial growth of mycelium of *T. asperellum*, *B. subtilis*, and Control (*Rhizoctonia* sp.). * Indicates that there were significant differences between the two microorganism and Control group (*p* < 0.01; Dunnett’s test).

From the results in Figure 1, significant differences were found between the control (*Rhizoctonia* sp.) and the treatments. The radial growth of the control was 3.34 cm, while that of *T. asperellum* was 1.01 and that of *B. subtilis* was 1.22 during 10 days. When analyzing the growth inhibition of the pathogen (Table 1), *T. asperellum* showed 69.5%, while in *B. subtilis*, it was 63.8%.

### 2.2. Determination of the Incubation Period of Endophytes on Roots of M. spicata Microplants

**Figure 2:**
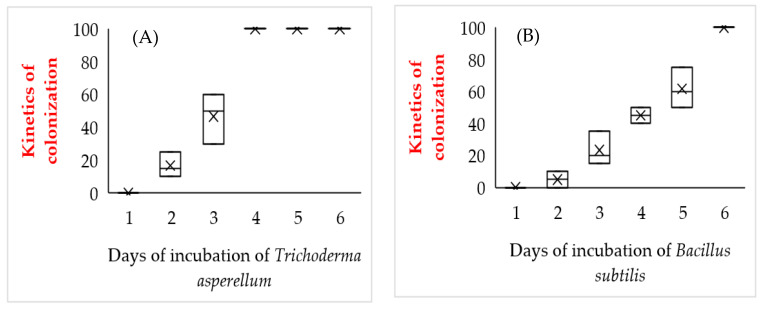
Kinetics of colonization. Kinetics of *Trichoderma asperellum* (**A**) and *Bacillus subtilis* (**B**), in *M. spicata* microplants.

According to the results shown in Figure 2, in vitro co-culture of *M. spicata* plantlets with both microorganisms resulted in endophytic colonization of the roots. In the case of *T. asperellum*, 100% colonization of root tissues was achieved from the fourth day of incubation. Plants inoculated with *B. subtilis* showed 100% root colonization after six days and competition for the culture medium was less aggressive compared to *T. asperellum*.

### 2.3. Evaluation of the Effect of Microplants Biotization on Survival during the Acclimatization Phase

In the present trial, the effect of biotization on microplants survival during the acclimatization phase was evaluated. The survival percentage of *M. spicata* plantlets was higher in the materials biotized with *T. asperellum* or *B. subtilis* with values of 95 to 93%, respectively, while plants without endophytes had a survival of 75%; as for plant height, a similar response was found where plants with endophytes presented heights between 12 and 15 cm, respectively, which were higher than the control that had an average height of 8 cm (Table 2).

### 2.4. Isolation of Endophytes in Tissues and Morphological and Molecular Identification of the Microorganisms under Greenhose Conditions

**Figure 3:**
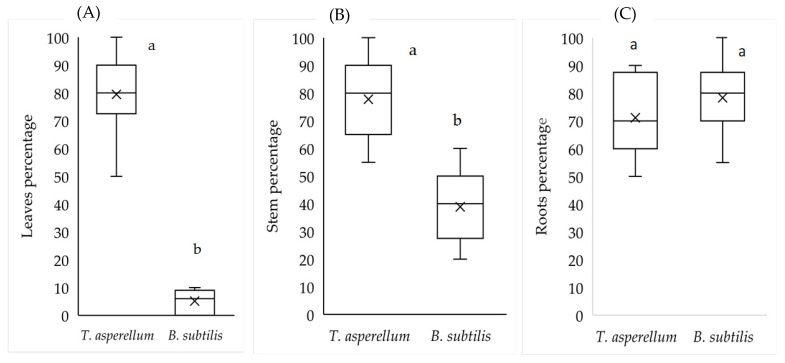
Isolation of endophytes in plants of *M. spicata* tissues. Percentage of leaf endohytes (**A**), root percentage (**B**), and stem tissues (**C**) with *T. asperellum* and *B. subtilis* endophytes in 30-day-old plants previously inoculated under in vitro conditions. Treatments not sharing the same letter(s) are statistically significant at *p* < 0.05; Tukey HSD.

The biotized plantlets were planted under nursery conditions for 30 days and the percentage of endophytic tissues was assessed. According to the results in Figure 3, it is shown that 80% of the leaf samples had the presence of *T. asperellum*, while *B. subtilis* was found in 5% of these tissues. In stems, *T. asperellum* was found in 77% of the tissues tested and *B. subtilis* was detected in 40%. In roots, the presence of both endophytes was demonstrated with values higher than 70%.

For the identification of micro-organisms, the isolations on potato, dextrose, agar (PDA) medium, at a temperature of 30 °C and after six days, showed the presence of ovoid unicellular structures similar to hyaline conidia on long, non-verticillate conidiophores. In the identification of the plates, the presence of septate, hyaline and thin mycelium was found. The conidiophores were branched with bottle-shaped, solitary or clustered phialides. Conidia are green, with a smooth or rough wall, typical characteristics of *T. asperellum*. Bacteria isolated on nutrient agar medium formed opaque cream-colored colonies and were gram-positive. When smeared on Petri dishes and stained with 5% malachite green for five minutes, the endospores turned into a light green color, characteristic of *Bacillus* bacteria. For molecular analysis, the identification name was determined from the best BLAST score obtained according to sequence identity and coverage. According to https://blast.ncbi.nlm.nih.gov/Blast.cgi (accessed on 13 May 2022), the sequences were shown to correspond to *Trichoderma asperellum* and *Bacillus subtilis*. These results confirmed that it was the endophyte initially inoculated on *M. spicata* microplants.

### 2.5. Effect of Biotizated M. spicata Plantlets on Dry Biomass and Incidence of Rhizoctonia sp. under Greenhouse Conditions

**Figure 4:**
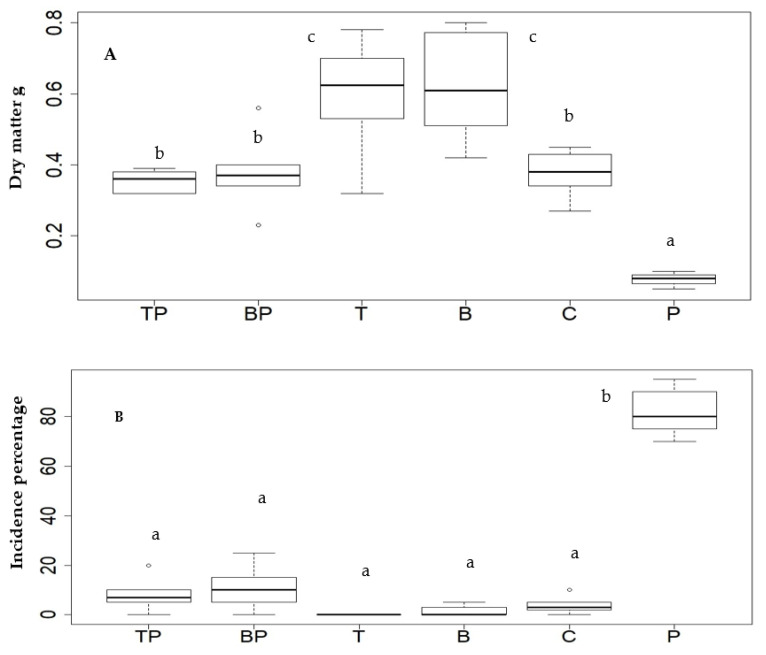
Effect of biotized *M. spicata* plantlets with *B. subtilis* and *T. asperellum*. Box and whisker plots on dry biomass (**A**) and the incidence of *Rhizoctonia* sp. (**B**). The treatments were: TP with endophyte *T. asperellum* + *Rhizoctonia* sp.; BP Plants with endophyte *B. subtilis* + *Rhizoctonia* sp.; T, mint plants with *T. asperellum* endophyte without pathogen; B. Plants with *B. subtilis* endophyte without pathogen; C, mint plants without endophyte (control); P, mint plants with *Rhizoctonia* sp. The data correspond to the means of 30 experimental units per treatment. Different letters indicate significant differences between treatments according to Tukey’s test at *p* < 0.05.

Plants with the highest biomass were those with *T. asperellum* and *B. subtilis* endophytes (Figure 4A). It was also shown that plants treated with the pathogen and without the endophyte (P) showed a lower biomass compared to the control not inoculated with the pathogen(C). In terms of pathogen incidence, a value of 82% was found in plants without the endophyte treated with *Rhizoctonia* (P), while in the other treatments, there were no significant differences. For example, in the TP and BP treatments, despite having the pathogen and endophyte, a relatively low incidence of the disease was achieved with values between 8 and 10%, respectively.

### 2.6. Effect of Biotizated M. spicata Plantlets on the Content of Some Specialized Metabolites under Nursery Conditions

**Figure 5:**
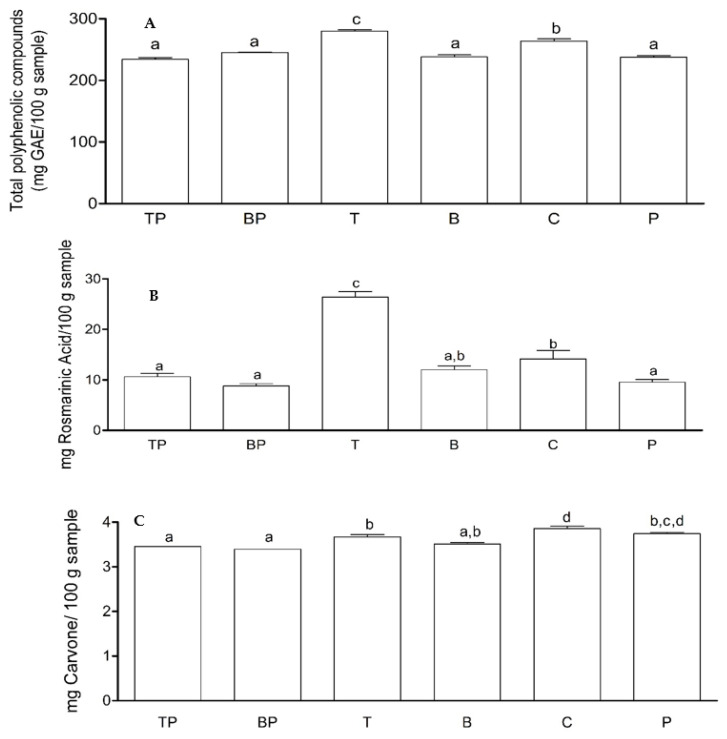
Contents of specialized plant metabolites in *M. spicata*. Rosmarinic acid content (**A**), total polyphenols (**B**), and carvone content (**C**) in samples of *M. spicata*. The treatments were: TP, Plants with endophyte *T. asperellum* + *Rhizoctonia* sp.; BP, Plants with endophyte *B. subtilis* + *Rhizoctonia* sp.; T, Mint plants with *T. asperellum* endophyte without pathogen; B. Mint plants with *B. subtilis* mint plants; C, mint plants without endophyte (control); P, mint plants without endophyte + *Rhizoctonia* sp. Treatments not sharing the same letter(s) are statistically significant at *p* < 0.05; Tukey HSD.

Regarding the content of total polyphenolic compounds (TPC), the treatment with the endophyte *T. asperellum* (T) showed the highest TPC content, with values of 280 mg GAE/100 followed by the control (C) with mg GAE/100 mg sample. The other treatments showed no significant differences (Figure 5A). The treatment with the endophyte *T. asperellum* (T) showed the highest rosmarinic acid (RA) content with a value of 28 mg RA/100 g sample. The treatments with the endophyte *B. subtilis* (B) and the control (C) did not differ significantly from each other, with values between 12 and 14 mg AR/100 g sample, respectively (Figure 5B). For carvone content, the treatments corresponding to the control (C) and with the pathogen without the endophyte (P) showed the highest carvone content with values between 3.9 and 3.8 mg carvone/100 g sample (Figure 5). When comparing the treatments with the pathogen (P) and the treatment with the endophytes *T. asperellum* (T) and with *B. subtilis*, no significant differences were found (Figure 5B). In recent years, research on plant–micro-organism association has been of great scientific and commercial interest due to its effect on improving plant quality, promoting plant development, and tolerance to biotic and abiotic problems [17].

One of the major phytosanitary problems in Mentha species is *Rhizoctonia* sp. whose control is difficult due to the formation of sclerotia which are resistance and survival structures. Therefore, agronomic cultural practices such as crop rotation and control of relative soil moisture are very important to combat this disease. In some cases, broad spectrum fungicides are used, but they are highly toxic and can accumulate in the tissues, which is not allowed in this type of crops of condiment and pharmaceutical interest.

Concerning in vitro antagonism, tests [18] demonstrated that endophytic microorganisms produce extracellular metabolites and cell-wall-degrading enzymes such as chitinase, β-1,3-glucanases and proteases, which could explain the results presented in Figure 1 where *T. asperellum* caused inhibition due to its enzymatic activity and competition for the culture medium. The antagonistic activity of *Bacillus* spp. may be due to the production of volatile organic agents such as hydrogen cyanide and antibiotics that inhibit the development of phytopathogenic fungi [19]. The results obtained coincide with those obtained by [20], who investigated some strains of *Bacillus* spp. that can inhibit the growth of various phytopathogens due to the production of subtilins and iturins that attack the cell wall of the fungi.

## 3. Discussion

Research on the biological control of *Rhizoctonia solani* on Mentha piperita showed that Pseudomonas fluorescens had the highest in vitro inhibition of the pathogen and was effective in reducing the disease under greenhouse conditions (23%), although T. viride and *B. subtilis* also reduced the incidence to 50% [21]. The main concern with the use of soil-applied biological control agents is the colonization and persistence of the microbial inoculum in plants due to abiotic factors such as temperature, humidity, soil conditions or biotic factors such as antagonism, competition, predation, among others that may reduce their efficacy under field conditions [22,23].

This study showed that biotization with endophytes on *M. spicata* microplants was effective, reducing the disease incidence to 8% for *T. asperellum* and 10% for *B. subtilis*, making it an alternative to conventional soil inoculation methods. According to [24,25], this method allows a better and faster interaction with the root system and a higher growth induction. It has been shown that although endophytes live in close association with plants, they need to overcome defense barriers such as specialized plant metabolites [26]; so, these microorganisms need to produce enzymes that break them down and can colonize tissues. In this context, knowledge of symbiotic mechanisms in the co-culture between endophytic microorganisms and cells, tissues and organs cultured in vitro, known as biotization, has gained importance [27].

However, one of the difficulties observed when inoculating *M. spicata* microplants with *T. asperellum* under in vitro conditions was the rapid colonization of the endophyte in the culture medium and necrosis of the plant collar; although, it was found that the plant survived until the sixth day, time that allowed the endophyte to associate with the plant.

The authors of [24] showed similar results in the in vitro inoculation of *Prunus* spp. rootstocks with the endophyte *T. harzianum*, where necrosis was observed in unrooted shoots after two to three days, while higher survival was obtained in rooted seedlings. *Trichoderma* spp. are opportunistic plant symbionts that can colonize the apoplast of plant roots, and then stems and leaves; [28] found that 24 h after inoculation with *Trichoderma* sp. in *Arabidopsis* roots, changes were observed in the transcriptome responding to different stimuli such as carbohydrates and chitin, as well as in genes involved in hormone biosynthesis and response to hormone signaling; [16] improved the survival of *Albizia amara* microplants biotized with *P. fluorescens* and *T. viride* up to 80%, while the control reached 5%.

Our results showed that microplants biotized with *T. asperellum* and *B. subtilis* significantly improved the biomass with respect to the control treatment (C), which produced remarkable advantages for the plants in the establishment processes and promoted a higher growth and survival of the microplants during the acclimatization phase. In fact, the use of endophytic microorganisms to promote plant growth has intensified significantly in recent years, as it is associated with effects such as better distribution of photoassimilates, phytohormone production, and nitrogen fixation, among others [29].

It was shown that plants biotized with the endophytes and then inoculated with *Rhizoctonia* sp. (TP and BP treatments) had a low incidence of the pathogen, compared to plants treated and infected only with the pathogen (P). This may be attributed to the fact that endophytic microorganisms inhabit similar ecological niches as the pathogen, which allows them to protect their environment and control it through competition, production of antagonistic substances, parasitizing directly or even prime plant defenses inducing resistance or tolerance [26]. Thus, both the host plant and the endophytes benefit from the interaction between them, with the endophyte helping the plant to stimulate its growth and development, and tolerance to biotic and abiotic stresses, for example, by reducing infection levels and suppressing pathogen growth or enhancing the solubilization of nutrients fixed in the soil [30].

Endophytic bacteria, such as *B. subtilis*, can protect plants by producing antibiotics that inhibit pathogen development and promote induced systemic resistance (ISR) [31]. Inoculation of endophyte microorganisms during micropropagation is an alternative that can improve crop quality and increase the content of specialized metabolites such as flavonoids and phenolic compounds in general [25,32,33].

Regarding the effect of biotization on specialized metabolites, it was observed that rosmarinic acid, which is a natural polyphenol found in many plants of the *Lamiaceae* family, was more than doubled in plants with the endophyte *T. asperellum* (28 mg RA/100 g sample) compared to control plants (12 mg RA/100 g sample). Rosmarinic acid is the main phenolic component of *Mentha* spp. [34], considered as a potent antiviral agent and with important antimicrobial effects [10]. Likewise, plants inoculated with this endophyte presented the highest production of total polyphenolic compounds that are biologically active and play a very important role in contributing to the antioxidant, sensory, astringency, and bitterness characteristics of the plant extract or essential oil [3]. In relation to the content of carvone, a monoterpene presents in most essential oils of *Mentha* spp. genus; it was shown that its concentrations were lower with endophytes and pathogen compared to control (C) plants, which could be explained by the alteration of the mevalonic acid metabolic pathway [35].

The effect of endophytes on plant metabolism is still under study, as some specialized metabolites can be produced by the combined activity of the microorganism and the host plant. *Bacillus* spp. have been found to serve as elicitors of metabolites such as ginsenosides in ginseng [36]; furthermore, these elicitors may directly participate in the transformation of compounds in plants and potentially promote the accumulation of active constituents in medicinal plants. However, the specific mechanisms regulating the shared metabolism are largely unknown [36]. It has been reported that colonization by endophytes can trigger defense responses of the host, one of these, the accumulation of anti-infective compounds is widely accepted as strategies against infections. In this way, the accumulation of anti-infective molecules could happen. Rosmarinic acid is the main phenolic component of *Mentha* spp. [33] considered as an anti-infective phenolic with antiviral and antimicrobial effects.

## 4. Materials and Methods

### 4.1. Chemicals and Reagents

All solvents were analytical and/or HPLC grade. Deionized water was obtained with a Milli-Q water purification system (Millipore, Bedford, MA, USA). The standard carvone was acquired from Sigma Aldrich (Buchs, Switzerland), and rosmarinic acid was purchased from the European Pharmacopoeia reference standard (Strasbourg, France). These standards were prepared as stock solutions in ethanol (1 mg/mL). For molecular analysis, we used nuclease-free water, magnesium chloride, and dNTPs acquired from Termo Fischer Scientific (Waltham, Massachusetts, USA); Buffer 10X, MyTaq from Meridian Bioscience; Backt F, R from IDT Integrated DNA Technologies.

### 4.2. Plant Material

Microplants shoots grown in MS culture medium [37] supplemented with 3% sucrose and 0.5 mg/L benzyladenine were used. Then, plantlets rooting was carried out under completely aseptic conditions in a culture medi-um composed of MS mineral salts added with sucrose (3%), indole butyric acid (0.5 mg/L), and agar-agar (0.7%); the internodes were planted in nine plastic boxes with 15 plantlets each and incubated at a temperature of 28 °C, with a photoperiod of 12 h light: 12 h darkness, with fluorescent lamps from Philips (Amsterdam, The Netherlands) light lamps (FFF 80 µmol m—2. S-1). Thirty days after sowing the plantlets formed roots [38].

### 4.3. Endophytic Microorganism

Strains of *T. asperellum* and *B. subtilis* from the collection of biological control agents of the company BIOQUIRAMA SAS^®^ (Antioquia, Colombia) were used, which are morphologically and molecularly characterized. *T. asperellum* fungus was cultivated in potato dextrose agar-acidified medium with lactic acid (25%) and incubated at 30 °C for seven days under dark conditions. Conidia were then recovered by scraping with a spatula and the agar surface washed with sterile distilled water and a drop of tween 20. The Conidia concentration was determined with a hemocytometer and suspensions were adjusted to 1.0E8 conidia/mL. The *B. subtilis* bacterium strain was incubated at 37 °C for 24 h in dark, and the appearance of colonies was examined and a suspension with a final concentration of 1.0E5 CFU/mL was prepared. To guarantee the quality of the inoculum, tests were carried out to evaluate the viability of each microorganism and germination was determined 24 h after inoculation in PDA.

### 4.4. Isolation of Rhizoctonia sp.

The pathogenic fungus *Rhizoctonia* sp. was obtained from root samples of plants with stem and rhizome lesions in commercial cultures of *M. spicata*. Isolations were made in PDA culture medium and the study of morphological characteristics such as mycelium, color, type of growth, presence of conidia, presence of septa was carried out for four days at 28 °C [39]. This process was carried out in the plant health laboratory of the Catholic University of the East.

### 4.5. Antagonism Tests T. asperellum and B. subtilis vs Rhizoctonia sp.

The growth rate of *T. asperellum*, *B. subtilis*, and *Rhizoctonia* sp. was determined by its radial growth rate in vitro in a PDA culture medium. The competition between *T. asperellum* and *Rhizoctonia* sp. was carried out in dual culture, for which a 5 mm diameter triangle of agar with mycelium of *Rhizoctonia* sp. was used. The strain was placed at the end of the Petri dish and another agar triangle with the *T. asperellum* strain was placed at the opposite end. Subsequently, the cultures were incubated at 30 ± 2 °C for 10 days and every two days, the radial growth of the mycelium of the fungal colony under study was calculated, calculating the mean percentage values of radial growth inhibition. On the other hand, in the confrontations of *Rhizoctonia* sp. with *B. subtilis*, the Petri dish was divided into two parts and a 5 mm diameter triangle of agar with mycelium of *Rhizoctonia* sp. was placed in one of the ends, and in the other half of the plate, *B. subtilis* was seeded by streaks on the entire surface of the medium; then, the cultures were incubated at 25 ± 1 °C for 10 days, each 2 days the radial growth diameter of the fungus *Rhizoctonia* sp. in the presence of the bacterial antagonist *B. subtilis* was measured.

The percentage of radial growth inhibition (PRGI) was determined by the formula used by [40]:

PRGI = [(R1 − R2) /R1] ∗ 100

where, R1 = largest radius (control pathogen) and R2 = minor radius (radius of the pathogen in confrontation with the antagonist).

### 4.6. Determination of the Incubation Period of the Endophytes in M. spicata Microplants

The purpose of this test was to determine the minimum time in which in vitro plants acquire the endophyte without microorganism competition for the culture medium without affecting the plantlets. The inoculum was prepared from a suspension of *T. asperellum* and *B. subtilis* spores, respectively, in sterile water in 2 mL microtubes. Spore concentration was determined using a Neubauer chamber and bacteria were quantified by serial dilution technique and colony count. Inoculations with the microorganisms consisted of adding a suspension of 2 mL of *T. asperellum* to the in vitro plantlets under aseptic conditions at a final concentration of 1.0E8 spores/mL and a 2 mL suspension of *B. subtilis* at a final concentration of 1.0E5 CFU/mL. The effect of the microorganisms on the plantlets and the determination of survival were evaluated daily. To verify that the in vitro plantlets of *M. spicata* had acquired the endophytes, root samples were taken daily, then disinfected with sodium hypochlorite (0.5%), and washed three times with sterile distilled water to remove excess disinfectants; the samples were then planted on acidified and non-acidified PDA media. For each treatment, three magenta vessels were used, each containing 15 microplants.

A randomized experiment with three repetitions was developed and each repetition consisted of 15 experimental units.

### 4.7. Evaluation of the Effect of Microplants Biotization on Survival during the Acclimatization Phase

Using the methodology mentioned above, in vitro rooted plantlets were inoculated with *T. asperellum* and left to incubate for four days, and with *B. subtilis*, the incubation was for six days. Microplants were transferred to a humid chamber and planted in seedlings trays with peat moss for a period of 30 days, after which a measurement of survival and growth of the seedlings was made. A randomized trial with 45 plants per treatment was used. Where the treatments corresponded to plants biotized with *T. asperellum*, plants biotized with *B. subtilis*, and a control of plantlets without endophytes.

### 4.8. Isolation of Endophytes in Tissues and Morphological and Molecular Identification of the Microorganisms under Greenhose Conditions

The plants hardened under greenhouse conditions were kept for 30 days in seed trays with sterilized peat moss. After that time, the plants were washed with running water and soap to remove the substrate particles. Then, the plants were disinfected with an iodine solution (1%), they were rinsed, and stem, leaf, and root fragments were selected and disinfected with ethanol (70%) for one minute and 2% sodium hypochlorite for five minutes. The different types of explants were incubated in a PDA culture medium acidified with lactic acid (25%) for fungi and PDA without acidification for bacteria. After 4 to 6 days of incubation at 30 °C, the presence of the microorganism in each of the tissues was evaluated, and the identification was made according to the morphological characteristics in the culture medium [41,42].

Molecular characterization of *Trichoderma* sp. DNA extraction was performed using the CTAB (cetyltrimethylammonium bromide) method (Doyle and Doyle [43]. The primers ITS-5 and ITS-U4 were used, which amplify a fragment of the eukaryotic 5.8S subunit rDNA:

▪ ITS5 (5′-GGAAGTAAAAGTCGTAACAAGG-3′) (White et al. [44]).
▪ ITS-U4 (5′-RGTTTCTTTTCCTCCGCTTA-3′) (Cheng et al. [45]).

PCR protocol with ITS primers:

For each PCR reaction, a final volume of 25 μL was used, containing nuclease-free water (15.8 μL), 10X PCR Buffer (2.5 μL), MgCl2 (1.5 μL), dNTPs (1 μL); ITS-5 (1 µL), ITS-U4 (1 µL), MyTaq (0.2 µL), and DNA (2 µL). The sample was taken to a Bio-Rad thermocycler (Techview, Singapore), for the PCR process, and initial denaturation was performed at 94 °C for 4 min, followed by 6 cycles of heating at 94 °C for 30 s, annealing at 60 °C for 40 s, and extension at 72 °C for one minute. After 33 cycles of heating at 94 °C for 30 s each one, alignment at 45 °C for 1 min, and extension at 72 °C for 2 min, the samples were subjected to a final extension cycle at 72 °C for 10 min.

Molecular characterization of *Bacillus* sp., for DNA extraction, the protocol proposed by Ribeiro et al. [46] was used with modifications that consisted of using a 200 µL tube containing *Bacillus* sp. in 50 µL of nuclease-free water; it was placed in a thermal cycler (T100 Thermal Cycler, BIORAD (Techview, Singapore), the incubation mode was selected, the sample was subjected to 90 °C for 5 min, and the PCR protocol was continued. For the amplification of the 16S segment, the sequences of the Backt F and R primers were used:

▪ Backt_341 F (5′-CCTACGGGNGGCWGCAG-3′)
▪ Backt_805 R (5′-GACTACHVGGGTATCTAATCC-3′)

For the preparation of the PCR mixture per reaction 15.8 µL of nuclease-free water, 1.5 µL of Magnesium Chloride, and 1 µL of Deoxynucleotides, 2.5 µL of 10X Buffer, plus 0.2 µL of MyTaq were added. A total of 1 µL Backt 341 F primer plus 1 µL Backt 805 R, 2 µL DNA extract was added for each reaction. For the PCR process, an initial denaturation was performed at 94 °C for 5 min, followed by 6 cycles of heating at 94 °C for 30 s, annealing at 55 °C for 1 min, and extension at 72 °C for 2 min. After 31 cycles of heating at 94 °C for 1 min, alignment at 45°C for 1 min, and extension at 72 °C for 2 min, the samples were finally subjected to a final extension cycle at 72 °C for 10 min.

For the reveal of the results in agarose gel, a 1.7% agarose gel (Agarose LE, Promega, Madison, WI, USA) was prepared. The electrophoresis chamber and seeding of the samples (PCR products) were mixed with the loading buffer (EZ-Vision, VWR Amresco (Solon, Ohio, USA) (loading buffer = 6 µL plus PCR product = 4 µL). A reaction for molecular weight markers (Smobio, Hsinchu, Taiwan) was included.

In the electrophoresis chamber (Bio-Rad Techview, Singapore), the agarose gel was subjected to 100 V, 400 mA for 40 min. The agarose gel was visualized in the transilluminator. It is required to consider the marking of the weight marker from 200 bp to 1000 bp. If any of the samples shows an amplification, a faint or intense band can be observed in a base pair measurement to be determined.

The obtained sequences were analyzed using the BLAST algorithm against the NCBI (National Center of Biotechnology Information, Bethesda, Maryland, USA) GenBank non-redundant nucleotide database to make a de novo identification. The tests were carried out on three plants and three repetitions were made for each tissue; then, the results were presented in percentages of tissues that showed the presence of the endophyte.

### 4.9. Evaluation of the Effect of Inoculation of Biotized Plants with Rhizoctonia sp. under Greenhouse Conditions

With the purpose of evaluating the effect of the biotized plants against the pathogenic agent *Rhizoctonia* sp., on the development of the plants (dry biomass), the incidence of the pathogen, and the expression of some metabolites, a test was carried out that consisted of using plants which conserved the endophyte corresponding to *T. asperellum* or *B. subtilis*, with or without the inoculation of the pathogen *Rhizoctonia* sp., a negative control inoculated with *Rhizoctonia* sp., and a control without endophyte or inoculation with the pathogen (Table 3). The plantlets were initially inoculated by immersing the slightly injured roots in a suspension of *Rhizoctonia* sp. for five minutes. Then, the seedlings were individually planted in plastic cups with 150 g of substrate made up of a mixture of sterile soil (50%), sand (25%), and peat (25%). Sixty days later, the incidence of the disease (% of living plants without symptoms), dry mass of the whole plants, and the presence of metabolites were determined. A random experimental design with 30 repetitions was carried out, where each experimental unit corresponded to a pot plant.

### 4.10. Determination of the Disease Incidence

Each plant was checked for the presence of disease symptoms and based on the total number of rhizomes; the percentage of incidence was estimated [6].

### 4.11. Determination of the Biomass of the Plants

Five plants were randomly selected from each treatment, the whole plants were weighed on an analytical balance (Metler Toledo, Polaris Parkway, Columbus, USA), and, then, they were placed in a MEMMERT stove (Schwabach, Germany) with a forced draft of air at 65 °C until constant weight.

### 4.12. Determination of Total Phenolic Content (TPC)

The TPC of each extract was determined by the Folin–Ciocalteu method. Gallic acid in a dynamic range of 10 to 100 μg/mL was used as a reference standard. A total of 25 μL of the extract was mixed with 125 μL of Folin–Ciocalteu reagent (1:10), both were diluted in distilled water. This was shaken and incubated at room temperature in the dark for 5 min, followed by the addition of 100 μL of Na_2_CO_3_ (7.5% w/v). After 60 min of incubation at room temperature and in the dark, absorbance reading was performed at 765 nm using a Synergy HT multi-mode microplate reader (Biotek instruments, Inc.; Winooski, Vermont, USA). The total polyphenol content was calculated by interpolation on a calibration curve performed with gallic acid. Results are expressed as milligram gallic acid equivalents per gram of sample (mg GAE/g sample).

### 4.13. Phenolic Compounds Identification by HPLC-DAD

HPLC chromatographic analysis was carried out on an Agilent 1200 series instrument (Agilent Technologies, Palo Alto, CA, USA), equipped with a vacuum degasser, an automatic autosampler, a quaternary pump and a diode array detector (DAD). Compound separation was performed using a Zorbax SB RRTH^®^ Santa Clara, CA, USA) C18 column (50 mm × 4.6 mm with 1.8 μm particle size) at 30 °C, with a flow rate of 1.0 mL/min. Once the separation methodology was optimized, the mobile phase consisted of 0.5% formic acid in water (A) and acetonitrile (B), and the used linear gradient was as follows: 0 min, 16% B; 4 min, 16% B; 8 min, 20% B; 11 min, 40% B; 12 min, 45% B; 13 min, 50% B; 14 min 60% B; 15 min, 16% B. The injection volume was 5 μL. The analyzed compounds were monitored in the DAD at 250 and 329 nm. For the quantification of rosmarinic acid in the samples, the external standard calibration method was used.

### 4.14. Gas Chromatography/Mass Spectrometry (GC-MS) Analysis

The acquisition of chromatograms and mass spectra was performed using an Agilent Technologies 7890 gas chromatograph (Wilmington, DE, USA) equipped with an ALS 7683B autosampler and MSD 5975C mass selective detector operating in electron ionization (EI) mode at 70 eV. The carrier gas used was Helium UAP 5.0 at 1 mL/min. The used column was a Zebron ZB 5Msi^®^ (30 m × 0.25 mm × 0.25 μm), and the system was calibrated with a C7-C40 n-alkane series (Sigma Aldrich 49452U; St. Louis, MO, USA). The temperature program for all separations was 2 min at 60 °C and 60 °C–260 °C at 5 °C/min. The injector temperature was 280 °C in splitless mode. Acquisition of spectra and chromatograms (SCAN) was performed using a single quadrupole mass selective detector (sQUAD) programmed at a temperature of 150 °C. The detector was tuned in automatic mode throughout all experiments.

GC/MS analysis was performed of the *M. spicata* obtained with the ethanol extraction solvent: water 80:20 in relation to the carvone majority compound. For the quantification of the carvone, the external standard calibration method was used. Subsequently, 1 µL of the samples was injected into the gas chromatograph. The obtained data were compared with the National Institute of Standards and Technology (NIST) 2017 commercial library. Agilent Masshunter Qualitative software (Santa Clara, CA, USA) was used for automatic deconvolution of the spectra and signal processing.

### 4.15. Statistical Analysis

The statistical package Wizard 4.3 was used for data analysis. In all cases, the assumptions of Lilliefors (Kolmogorov–Smirnov), Shapiro–Wilk, and Levine’s homogeneity of variance were checked. Once these assumptions were verified, an analysis of variance and Tukey’s test using the honest significant difference (HSD) were used to establish statistical differences between treatments. The confidence level used for the ANOVA analysis was 95%.

## 5. Conclusions

It was demonstrated that co-cultivation of *M. spicata* microplants with the endophytes *T. asperellum* and *B. subtilis* in the rooting phase allowed the biotization of 100% of the root tissues and had a positive impact on the improvement of plantlets survival during the acclimation phase with values between 95% to 93%, which is an alternative to reduce the high plant material losses in the hardening processes of micropropagation. Under greenhouse conditions, plants biotized with the two microorganisms showed the highest percentages of dry biomass and when inoculated with *Rhizoctonia* sp. the incidence of the pathogen was between 8 and 10%, compared to 82% in plants inoculated without the endophytes. As for the content of some specialized metabolites, the plants biotized with *T. asperellum* showed the highest contents of total polyphenolic compounds, with values of 280 mg/GAE/100 and of rosmarinic acid with values of 28 mg/100 g of sample, doubling the content of this phenolic compound compared to the control plants. The results show the potential of inoculation of *M. spicata* microplants with endophytes to improve seedling survival during the acclimation phase and subsequently under greenhouse conditions to improve growth, disease tolerance and increase the production of specialized metabolites. 

## Figures and Tables

**Table 1:** Inhibition of the *Rhizoctonia* sp. growth by the microorganisms *T. asperellum* and *B. subtilis* under in vitro conditions.

Microorganism	Growth Inhibition (%)
*T. asperellum*	69.5
*B. subtilis*	63.8

There were no significant differences at *p* < 0.05; Tukey HSD.

**Table 2:** Survival percentage and growth of microplants of *M. spicata* biotized with *T. asperellum* and *B. subtilis* during the acclimatization phase.

Treatments	Survival Percentage	Plant Height (cm)
*T. asperellum*	95a	12a
*B. subtilis*	93a	15a
Control	75b	8b

The data correspond to the means of 45 experimental units per treatment. Treatments not sharing the same letter(s) are statistically significant at *p* < 0.05; Tukey HSD.

**Table 3:** Treatments used for the evaluation of the effect of *Mentha spicata* plants with endophytes of *B. subtilis* and *T. asperellum* and the pathogen *Rhizoctonia* sp., on the incidence of the disease, dry mass (aerial part and roots), and specialized metabolites.

Code	Treatment	Code	Treatment
(TP)	Spearmint plants with endophyte *Trichoderma asperellum* + *Rhizoctonia* sp.	(B)	Spearmint plants with endophyte *Bacillus subtilis Rhizoctonia* free
(BP)	Spearmint plants with endophyte *Bacillus subtilis* + *Rhizoctonia* sp.	(C)	Spearmint plants without endophyte (test)
(T)	Spearmint plants with endophyte *T. asperellum Rhizoctonia* free	(P)	Spearmint plants infected with *Rhizoctonia* without endophyte

## Data Availability

Not applicable.
